# The Landscape of Genomic Imprinting at the Porcine *SGCE*/*PEG10* Locus from Methylome and Transcriptome of Parthenogenetic Embryos

**DOI:** 10.1534/g3.120.401425

**Published:** 2020-09-02

**Authors:** Jinsoo Ahn, In-Sul Hwang, Mi-Ryung Park, In-Cheol Cho, Seongsoo Hwang, Kichoon Lee

**Affiliations:** * Functional Genomics Laboratory, Department of Animal Sciences, The Ohio State University, Columbus, OH 43210; † Animal Biotechnology Division, National Institute of Animal Science, Rural Development Administration, Jeonbuk 55365, Republic of Korea; ‡ National Institute of Animal Science, Rural Development Administration, Jeju, 690-150, Republic of Korea

**Keywords:** genomic imprinting, *SGCE*, *PEG10*, porcine, parthenogenetic, embryo, whole-genome bisulfite sequencing, RNA sequencing, differentially methylated region

## Abstract

In mammals, imprinted genes often exist in the form of clusters in specific chromosome regions. However, in pigs, genomic imprinting of a relatively few genes and clusters has been identified, and genes within or adjacent to putative imprinted clusters need to be investigated including those at the *SGCE*/*PEG10* locus. The objective of this study was to, using porcine parthenogenetic embryos, investigate imprinting status of genes within the genomic region spans between the *COL1A2* and *ASB4* genes in chromosome 9. Whole-genome bisulfite sequencing (WGBS) and RNA sequencing (RNA-seq) were conducted with normal and parthenogenetic embryos, and methylome and transcriptome were analyzed. As a result, differentially methylated regions (DMRs) between the embryos were identified, and parental allele-specific expressions of the *SGCE* and *PEG10* genes were verified. The pig imprinted interval was limited between *SGCE* and *PEG10*, since both the *COL1A2* and *CASD1* genes at the centromere-proximal region and the genes between *PPP1R9A* and *ASB4* toward the telomere were non-imprinted and biallelically expressed. Consequently, our combining analyses of methylome, transcriptome, and informative polymorphisms revealed the boundary of imprinting cluster at the *SGCE*/*PEG10* locus in pig chromosome 9 and consolidated the landscape of genomic imprinting in pigs.

In mammals, imprinted genes often exist in the form of clusters in specific chromosome regions (Lewis and Reik 2006). Therefore, detailed comparative characterization of large imprinted clusters is important to understand epigenetic regulation of genomic imprinting. In pigs, progress has been made in identifying imprinted genes (Li *et al.* 2008; Bischoff *et al.* 2009; Oczkowicz *et al.* 2012; Tian 2014; Ahn *et al.* 2020); however, still a relatively few genes and clusters have been identified and genes within or adjacent to putative imprinted clusters need to be investigated, including those at the *SGCE*/*PEG10* locus.

In the mouse, a large cluster of imprinted genes within a 1-Mb region between *Col1a2* and *Asb4* in proximal (centromeric) chromosome 6 (6A1) was previously reported (Ono *et al.* 2003). Of nine murine genes belonging to the above interval (*Col1a2*, *Casd1*, *Sgce*, *Peg10*, *Ppp1r9a*, *Pon1*, *Pon3*, *Pon2*, and *Asb4*), two genes, *Sgce* and *Peg10*, are paternally expressed imprinted genes (Piras *et al.* 2000; Ono *et al.* 2003). This paternal expression appears critical in mammalian development as evidenced by early embryonic lethality of mice with maternal duplication of the proximal chromosome 6 cluster and with a deficiency of *Peg10* (Ono *et al.* 2006). Three genes (*Ppp1r9a*, *Pon3*, and *Pon2*) from the same cluster were maternally expressed in the embryonic stages with some exceptions, and the *Asb4* gene showed a strong maternal expression (Mizuno *et al.* 2002; Ono *et al.* 2003). Expressions of *Col1a2* and *Casd1* were biallelic, and *Pon1* expression was low in the mouse embryo and undetectable in human-derived cell lines (Mizuno *et al.* 2002; Okita *et al.* 2003; Ono *et al.* 2003). Although the way that the epigenetic germline mark affects the expression of genes in *cis* remains to be determined, a single *cis*-acting imprinting control region (ICR) which may establish and maintain imprinting throughout the *Col1a2-Asb4* cluster was identified at the *SGCE*/*PEG10* differentially methylated region (DMR) (Ono *et al.* 2003; Monk *et al.* 2008). This DMR/ICR acquired allele-specific DNA methylation in the maternal germline. In humans, on chromosome 7 (7q21.3), paternal expressions of *SGCE* and *PEG10* was conserved (Ono *et al.* 2001; Müller *et al.* 2002; Grabowski *et al.* 2003). Human *PPP1R9A* gene was found to be imprinted and maternally expressed mainly in skeletal muscle tissues (Nakabayashi *et al.* 2004). In ovine fetuses, a putative imprinted interval was reported to be extended to between *CASD1* (maternally expressed) and *PON3* (paternally expressed), while the paternal expression of *SGCE* and *PEG10* and maternal expression of *PPP1R9A* were conserved (Duan *et al.* 2018).

In this study, the imprinted cluster containing the *SGCE*/*PEG10* locus was determined by comparing parthenogenetic (PA) and normal control (CN) porcine embryos using whole-genome bisulfite sequencing (WGBS) and RNA sequencing (RNA-seq). In addition, to confirm the expression pattern of *PPP1R9A*, a single nucleotide polymorphism-based assay was performed. We report that the orthologous pig imprinted interval was between *SGCE* and *PEG10*, since both the centromere-proximal genes (*COL1A2* and *CASD1*) and the telomeric genes between *PPP1R9A* and *ASB4* were non-imprinted and biallelically expressed.

## Materials And Methods

### Ethics statement

All the treatments and experiments related to pigs were performed in accordance with study protocols and standard operating procedures that were approved by the Institutional Animal Care and Use Committee of the National Institute of Animal Science, Rural Development Administration (RDA) of Korea (approval number NIAS2015-670).

### In vitro maturation of porcine oocytes and production of parthenogenetic embryos

Pig oocytes were collected and *in vitro* maturation (IVM) was performed as described in our previous reports (Kwon *et al.* 2017; Ahn *et al.* 2020). In brief, from a local slaughterhouse (Nonghyup Moguchon, Gimje, Korea), ovaries from Landrace x Yorkshire x Duroc (LYD) pigs were obtained, and then immediately transported to our laboratory in saline solution supplemented with penicillin and maintained at 30–35° in a thermos. After collection of cumulus-oocyte complexes (COCs), they were washed in Tyrode’s lactate-Hepes containing 0.1% (w/v) polyvinyl alcohol. Oocytes surrounded by several layers of cumulus cells were collected and washed three times in TCM-199 (GIBCO, Grand Island, NY, USA) which is supplemented with 0.1% polyvinyl alcohol (w/v), 3.05 mM D-glucose, 0.91 mM sodium pyruvate, 0.57 mM cysteine, 0.5 µg/ml luteinizing hormone, 0.5 µg/ml follicle stimulating hormone, 10 ng/ml epidermal growth factor, 10% porcine follicular fluid (pFF), 75 µg/ml penicillin G, and 50 µg/ml streptomycin. For IVM, in each well of five four-well dishes (Nunc, Roskilde, Denmark) containing 500 µL of maturation medium, 50 COCs were placed and the oocytes were matured for 40–42 h at 38.5° in an incubator containing 5% CO_2_. Cumulus cells were then removed, and oocytes having the first polar body were selected and activated. In a fusion chamber with 250 µm diameter wire electrodes (BLS, Budapest, Hungary) covered with 0.3 M mannitol solution containing 0.1 mM MgSO_4_, 1.0 mM CaCl_2_, and 0.5 mM Hepes, selected oocytes were placed and fusion was achieved by two DC pulses (1 sec interval) of 1.2 kV/cm for 30 µs using an LF101 Electro Cell Fusion Generator (Nepa Gene Co., Ltd., Chiba, Japan). After electric stimulation of oocytes followed by 2 h of stabilization period, 200 parthenogenetic embryos were placed into oviducts of each of the two LYD surrogate gilts aged 12 months at onset of estrus (*i.e.*, a total of 400 parthenogenetic embryos for two surrogates) to produce developed parthenogenetic embryos.

### Collection of both fertilized control (CN) embryos and parthenogenetic (PA) embryos

To produce fertilized control (CN) embryos, during the natural heat period at the onset of estrus (day 0) two LYD gilts were naturally mated with boars twice with a 6 h interval. Both 12 CN embryos and six PA embryos were recovered at day 21 from the gilts and surrogates, respectively, that had been confirmed pregnant by an ultrasound examination. Regarding the recovery, it was considered that some imprinted genes in pigs (*e.g.*, *H19*, *IGF2*, *XIST* and *PEG10*) does not exhibit monoallelic expression until the blastocyst stage (Park *et al.* 2011), and porcine parthenogenetic embryos undergo morphological changes at approximately day 30 of gestation (Bischoff *et al.* 2010) and ceased fetal development with degenerated organs having a high rate of apoptosis of cells at day 35 of gestation (Moss *et al.* 2020). After the gilts and surrogates were killed, their reproductive tracts were sectioned and placenta were isolated from the uterus. Then, embryos separated from the surrounding placenta were placed on a piece of cleaning tissue in order to dry liquid on the surface of the embryos. Morphologically intact embryos with comparable sizes were selected for further experiments (lengths of 2.2, 2.1, and 2.1 cm for CN and 2.0, 2.0, and 1.6 cm for PA), and stored in liquid nitrogen until further use.

### Genomic DNA isolation and whole-genome bisulfite sequencing (WGBS) analysis

From the whole collected embryos of CN and PA (n = 3 for each), genomic DNA was isolated and fragmented. For optimization of bisulfite conversion of genomic DNA, Accel-NGS Methyl-Seq DNA Library Kit (Swift Biosciences, Inc. Ann Arbor, MI, USA) was used according to the manufacturer`s instructions. PCR was conducted with adapter primers and Diastar EF-Taq DNA polymerase (Solgent, Daejeon, Korea) to amplify the bisulfite-treated DNA, under the following thermal conditions: 3 min at 95° followed by 35 cycles of 30 sec at 95°, 30 sec at 60°, and 30 sec at 72°, and a final extension for 5 min at 72°. After bead-based clean-up, the PCR products were sequenced using HiSeqX sequencer operated by Macrogen (Seoul, Korea). For each of the WGBS libraries, the number of raw reads were approximately 848M (CN1), 864M (CN2), 868M (CN3), 842M (PA1), 859M (PA2), and 851M (PA3). After trimming adapter sequences and filtering out reads shorter than 20bp using Trim Galore (v0.4.5), cleaned reads for each library [approximately 847M (CN1), 862M (CN2), 866M (CN3), 840M (PA1), 857M (PA2), and 849M (PA3)] were retrieved (Table S1). After mapping to the reference genome (Sscrofa11.1/susScr11) with BSMAP (v2.87), the clean mapped reads were sorted and indexed. Using ‘methylatio.py’ script in BSMAP, extraction of the methylation ratio of every single cytosine from the mapping results was conducted. DMRs were identified by the program metilene (v0.2-8) (Juhling *et al.* 2016) using the methylation ratios at all CpGs. The criteria for DMR were a presence of more than 10 CpGs, a genomic distance of less than 300 bp between CpGs, and a mean methylation difference of more than 0.2 between groups. DMRs with false discovery rate (FDR) < 0.05 were obtained as significant DMRs (Table S2). To visualize methylation ratios and significant DMRs throughout the genomic coordinates of the imprinted cluster, the R/Bioconductor package Gviz (v1.28.3) (Hahne and Ivanek 2016) was used.

### RNA extraction, cDNA library construction, and RNA sequencing (RNA-seq)

Total RNA from whole CN embryos (n = 3) and whole PA embryos (n = 3) samples was isolated with TRIzol reagent (Sigma-Aldrich, USA) following the manufacturer’s instructions. DNase I was treated to the RNA samples to avoid genomic DNA contamination. The RNA samples were electrophoresed in 1.2% agarose gels to evaluate the integrity of RNA, which was then confirmed by more than 2 of 28S/18S rRNA ratio and more than 7 of RNA integrity number (RIN) using an Agilent 2100 BioAnalyzer. Using the ratios of A260/A280 and A260/A230 (1.8–2.0), the concentrations of RNA were assessed. One ug of total RNA was used to construct cDNA libraries with the TruSeq RNA Sample Prep Kit v.2 (Illumina, San Diego, CA, USA). The final cDNA library was produced using the protocol consisted of polyA-selected RNA extraction, RNA fragmentation, random hexamer primed reverse transcription and amplification. Quantification of the cDNA libraries was done by quantitative Real-Time PCR (qPCR) according to the qPCR Quantification Protocol Guide, and qualification of the libraries was assessed using the Agilent 2100 Bioanalyzer. The Illumina HiSeq2500 RNA-seq platform was used to sequence the library products (100nt paired-end). The number of raw reads generated for each RNA-seq library were approximately 77.3M (CN1), 73.5M (CN2), 77.7M (CN3), 80.7M (PA1), 79.9M (PA2), and 81.0M (PA3). After adapter trimming and quality filtering, the number of remaining cleaned reads were approximately 76.8M (CN1), 73.0M (CN2), 77.2M (CN3), 80.0M (PA1), 79.3M (PA2), and 80.3M (PA3) (Table S1). From the Assembly database at the National Center for Biotechnology Information (NCBI) (http://www.ncbi.nlm.nih.gov/assembly/GCF_000003025.6), the reference genome sequence of *Sus scrofa* (Sscrofa11.1/susScr11) and annotation files were downloaded. Then, using HISAT2 (v2.1.0) (Kim *et al.* 2015) with default parameter settings, the clean sequencing reads were aligned to the reference genome, except for the parameter–dta regarding tailored transcriptome assembly. BAM format containing RNA-seq alignments were produced and sorted by SAMtools (v1.9), and then BAM file-derived read coverage (or depth) was plotted and visualized using the R/Bioconductor package Gviz (v1.28.3) (Hahne and Ivanek 2016).

### Analysis of differential gene expression

The htseq-count script (Anders *et al.* 2015) was used to count aligned RNA-seq reads using each BAM file and an annotation file as inputs, and a count matrix was generated. Using the R/Bioconductor package DESeq2 (v1.26.0) (Love *et al.* 2014), read counts from individual fetal samples were normalized for sequencing depth and differential expression between CN embryos and PA embryos was calculated. To obtain significant differentially expressed genes (DEGs), the combined criteria of FDR < 0.05 and the absolute log2-fold change > 1 was used, where a fold change is defined as the expression in samples of PA divided by the expression in samples of CN.

### Polymorphism-based assays

Potential single nucleotide polymorphism (SNP) sites on the last exon of the porcine *PPP1R9A* gene was obtained from the Ensembl Genome Browser (http://www.ensembl.org). The gDNAs of boar (Korean native pig, KNP), sow (KNP), and offspring (neonates, 2-day-old) were isolated from ear tissue. RNA was extracted from six tissues (heart, kidney, liver, lung, muscle, and spleen) from neonates (2-day-old) and reverse-transcribed to cDNA. A primer set for amplification of a genomic DNA (gDNA) fragment containing those potential SNPs (316 bp) was designed on the last exon: forward, 5′-AGGCTCTTGGGATGACATCAT-3′ and reverse, 5′-ACGGTTCCCCAG TCTGCCAAAG -3′. To amplify a corresponding cDNA fragment (350 bp) while avoiding genomic DNA contamination, a forward primer (5′-ACTGGAGAGCAACTCCTGCAGT-3′) was designed on the exon coming just before the last exon and was coupled with the above reverse primer on the last exon. The conditions for PCR amplification were 95° for 2 min, appropriate cycles with linear amplification ranges of 95° for 20 s, 60° (gDNA) or 54° (cDNA) for 40 s, 72° for 30 s, with an additional extension step at 72° for 5 min. The amplified gDNA fragment was sequenced to identify heterozygous offspring with potential SNPs. The informative SNP sequences in gDNA of offspring were compared with sequences in amplified cDNA of offspring to determine maternal and/or paternal expression. Those SNP sequences of gDNA in the boars and sows were compared with above sequences to determine parent-of-origin-specific gene expression.

### Data availability

The WGBS and RNA-seq datasets generated in this study have been submitted to the NCBI BioProject database under accession number PRJNA657886 (https://www.ncbi.nlm.nih.gov/bioproject/PRJNA657886) and PRJNA658544 (https://www.ncbi.nlm.nih.gov/bioproject/PRJNA658544), respectively. DMRs called by the metilene software are included in Table S2. Supplemental material available at figshare: https://doi.org/10.25387/g3.12845840.

## Results

### Differentially methylated regions were only located at the SGCE/PEG10 locus within the COL1A2-ASB4 region in pigs

Nine genes (from *COL1A2* to *ASB4*) in pig chromosome 9 were mapped to an approximate 1-Mb region between 74.17 and 75.20 Mb according to the genome sequence of *Sus scrofa* (Sscrofa11.1/ susScr11). The order of those genes in this locus is conserved in the human chromosome 7 (7q21.3) and mouse chromosome 6 (6 A1). In the mouse, DNA methylation in this region was examined in four CpG islands located in the promoter regions of the *Casd1*, *Sgce-Peg10*, *Ppp1r9a*, and *Pon2* genes, and bisulfite sequencing analyses between reciprocal F1 hybrids revealed that only the promoter region of *Sgce-Peg10* is differentially methylated in a parent-of-origin-specific manner (maternally hypermethylated) and the other three regions were non-methylated (Ono *et al.* 2003).

**Figure 1:**
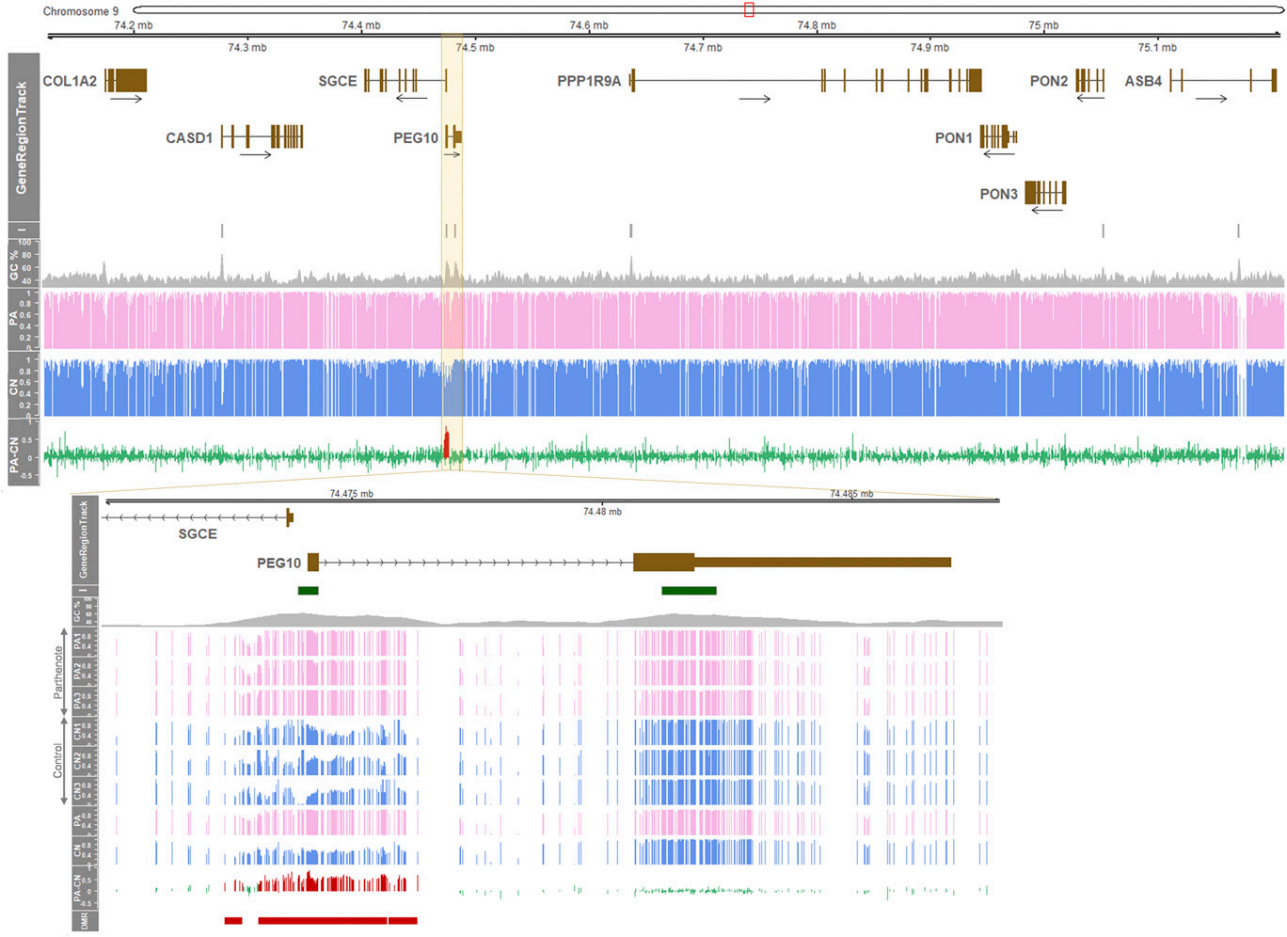
High-throughput DNA methylation profiling of the *SGCE*/*PEG10* locus in porcine embryos determined by WGBS. GeneRegionTrack represents an approximate 1.03-Mb region between the *COL1A2* and *ASB4* genes on porcine chromosome 9 (chr9:74,174,484 – 75,203,450). Nine genes are indicated by brown boxes for protein-coding transcripts (short boxes: noncoding region; tall boxes: protein-coding region), and directions are marked by black horizontal arrows. *From I track to PA-CN track*: *I*, CpG islands; *GC%*, GC content; *PA*, mean methylation ratio of parthenotes (pink histogram lines); *CN*, mean methylation ratio of controls (blue histogram lines); and *PA-CN*, mean methylation ratio of parthenotes subtracted by that of controls (green histogram lines). In PA-CN track, hypermethylated DMR in PA (FDR < 0.05) is overlaid with red histogram lines. *Yellow highlighted area*, an area containing the promoter regions of *SGCE* and *PEG10* and two CpG islands that is zoomed in. In the bottom plot, *PA1 - 3*, methylation ratios of three different parthenotes; *CN1 - 3*, methylation ratios of three different controls; *PA*, mean ratio; *CN*, mean ratio; *PA-CN*, PA subtracted by CN; and *DMR*, differentially methylated region (FDR < 0.05).

Similarly, in pigs, the promoter regions of the *CASD1*, *SGCE-PEG10*, *PPP1R9A*, and *PON2* genes contained CpG islands, and only the areas containing the promoter regions of *SGCE* and *PEG10* displayed DMR (Figure 1). DNA methylation of each of the three parthenotes and three controls was exhibited, and there were two CpG islands: one located in the 1^st^ exons and intergenic region of *SGCE* and *PEG10* and the other located in the 2^nd^ exon of *PEG10* (Figure 1). The genomic DNA around the latter CpG island was hypermethylated in all CN and PA embryos indicating non-DMR; whereas, hypomethylation of CN was found at the 1^st^ exons of *SGCE* and *PEG10*, intergenic region, and introns where GC content is high (Figure 1). As a result, maternally hypermethylated DMR in PA was identified.

### Promoter regions of genes in the COL1A2-ASB4 region mostly showed non-methylation

**Figure 2:**
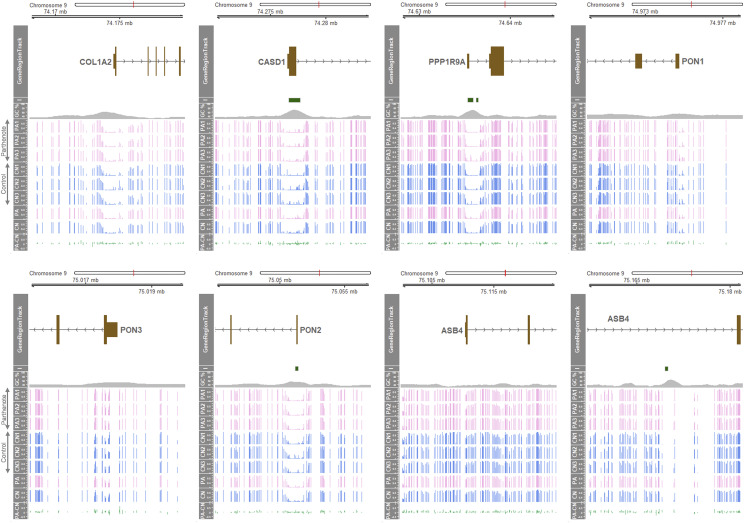
Promoter regions of the seven genes other than *SGCE* and *PEG10* and CpG islands in the *COL1A2-ASB4* region and methylation profiles. GeneRegionTrack represents regions containing a transcription start site and the first exon of the seven genes. *From I track to PA-CN track*: *I*, CpG islands; *GC%*, GC content; *PA1 - 3*, methylation ratios of three different parthenotes; *CN1 - 3*, methylation ratios of three different controls; *PA*, mean methylation ratio of parthenotes; *CN*, mean methylation ratio of controls; and *PA-CN*, mean methylation ratio of parthenotes subtracted by that of controls.

A close view of promoter regions of seven genes other than *SGCE* and *PEG10* was obtained. The promoter regions close to transcription start sites and first exons of the *CASD1*, *PPP1R9A*, and *PON2* genes enclosed CpG islands and high GC contents. These promoter regions were almost non-methylated in both CN and PA as shown by a regional hypomethylation (Figure 2). It suggests that these regions are biallelically active and the *CASD1*, *PPP1R9A* and *PON2* genes are not imprinted. The promoter region of the *COL1A2* gene also showed a similar hypomethylation, suggesting active and non-imprinting status of this gene (Figure 2). In addition, the promoter region of the *PON1* gene contained a relatively narrow range of hypomethylation and the promoter regions of the *PON3* and *ASB4* genes did not possess hypomethylated areas in both CN and PA, suggesting a relatively inactive status of these promoters and non-imprinting of these genes (Figure 2)

### Allele-specific expression in the porcine COL1A2-ASB4 region showed a partly conserved pattern

**Figure 3:**
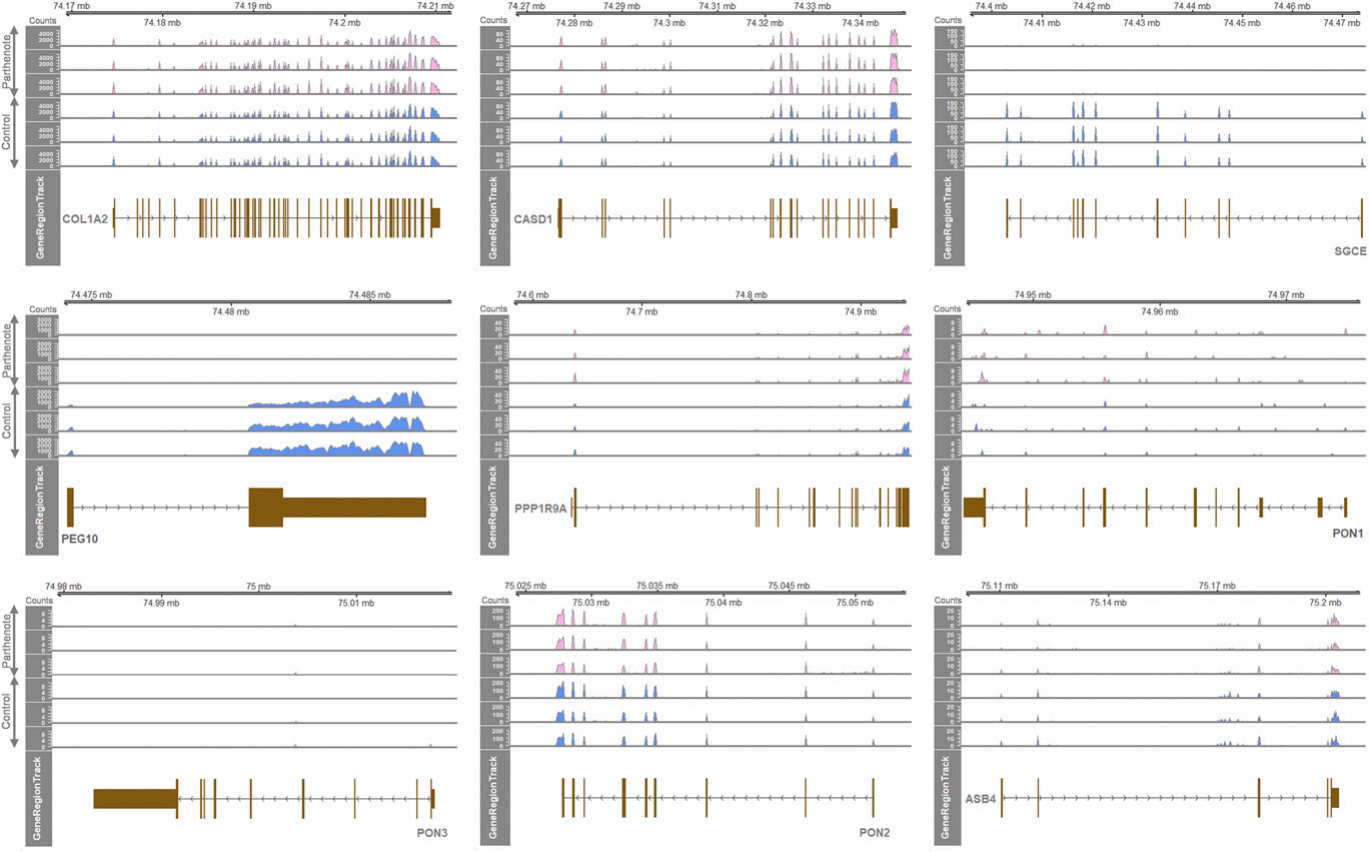
Allelic gene expression in porcine *SGCE/PEG10* locus. Nine genes in the porcine *COL1A2-ASB4* region are displayed in a gene order (from top left to bottom right). The RNA-seq read coverages, or depths, extracted from BAM files are shown as counts (Y axis) in each track of PA embryo (n = 3) and CN embryo (n = 3). PA, parthenote; CN, control.

Expression levels of each gene in the porcine *COL1A2-ASB4* region were investigated with RNA-seq analysis. The *SGCE* and *PEG10* genes were expressed only in CN, but not in PA (Figure 3). Because CN has one paternal and one maternal allele and PA has two maternal alleles, the sole expression in CN, but not in PA, could be derived from expression in the paternal allele (‘paternal expression’) which is consistent with previous reports (Bischoff *et al.* 2009; Park *et al.* 2011). Among the genes between *PPP1R9A* and *ASB4* toward the telomere, *PON2* was expressed biallelically as described previously (Zhou *et al.* 2011) and expression of *ASB4* was also biallelic (Figure 3). Expression of *PON1* was low, but appeared to be biallelic, and *PON3* expression was not detectable. Regarding expression of *PPP1R9A*, previous studies showed that it is expressed biallelically in tissues from 2-month piglets, but maternally in placental tissues (Bischoff *et al.* 2009; Zhang *et al.* 2011), and it appeared to be biallelic (Figure 3). In addition, expressions of both the *COL1A2* and *CASD1* genes at the centromere-proximal region were biallelic. Taken together, compared with humans and mice, only *SGCE* and *PEG10* genes showed a conserved paternal allele-specific expression pattern.

**Figure 4:**
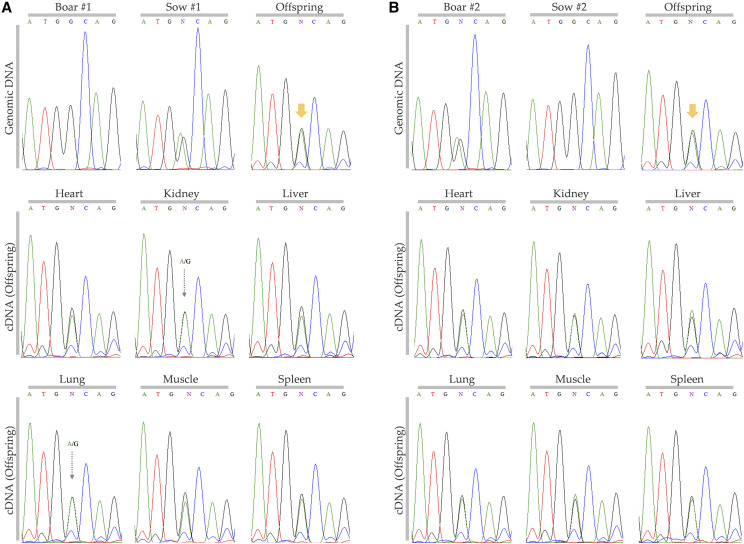
Polymorphism-based analysis of parent-of-origin dependent allele-specific expression of the porcine *PPP1R9A* gene. Amplified gDNA and cDNA fragments containing an informative SNP (rs329892462) in the last exon of *PPP1R9A* gene were sequenced. The downward arrows (in orange) point out the informative (heterozygous) SNP in gDNA of offspring for which cDNA of offspring was examined. Two different combinations of boar and sow and their offspring were analyzed (A and B). All cDNA from multiple tissues of offspring showed biallelic expression of A/G at the SNP site.

To validate the above expression patterns, DEGs were screened. Among the nine genes in the porcine *COL1A2-ASB4* region, the *SGCE* and *PEG10* genes were differentially expressed and showed significantly higher expression in CN indicating their paternal allele-specific expression (Table 1). In contrast, the expression of *PPP1R9A* tended to be higher in PA, but it was statistically nonsignificant indicating its expression is biallelic. To confirm the expression pattern of *PPP1R9A*, a SNP-based assay was performed as presented below (Figure 4). The rest of detected genes, the *COL1A2*, *CASD1*, *PON1*, *PON2*, and *ASB4* genes, did not show a difference between CN and PA indicating their biallelic expression and expression of the *PON3* gene was not detected (Table 1).

**Table 1:** Analysis of differentially expressed genes between parthenogenetic and control embryos

ID	Gene	M.Count^a^ (CN)	M.Count (PA)	log2FoldChange (PA/CN)	P-Value	P.adj^b^
ENSSSCG00000015326	*COL1A2*	60178.33	61307.49	0.03	0.80	0.94
ENSSSCG00000015327	*CASD1*	1433.21	1551.01	0.11	0.32	0.65
ENSSSCG00000015328	*SGCE*	1426.96	35.66	−5.32	2.01E-25	3.97E-23
ENSSSCG00000036049	*PEG10*	40529.77	27.35	−10.55	0.00E+00^c^	0.00E+00
ENSSSCG00000015329	*PPP1R9A*	586.36	733.52	0.32	0.10	0.38
ENSSSCG00000015332	*PON1*	72.21	76.73	0.09	0.77	0.92
ENSSSCG00000029515	*PON3*	ND	ND	ND	ND	ND
ENSSSCG00000015331	*PON2*	895.78	855.09	−0.07	0.69	0.89
ENSSSCG00000015333	*ASB4*	109.79	83.32	−0.40	0.13	0.43

Test for differentially expressed genes was conducted by the R/Bioconductor package DESeq2. ^a^Mean of read counts which is presented as mean ± SEM ^b^Benjamini–Hochberg (BH) adjusted *p*-value. ^c^Value of 0.00E+00 represents that the significance was not measurable because it reached a maximum significance set by the program. *ND*, not detectable.

### SNP-based analysis revealed biallelic expression of PPP1R9A

Among SNPs in the last exon of the porcine *PPP1R9A* gene, a SNP rs329892462 (G/A) was heterozygous (informative) in our gDNA samples from offspring (Figure 4). The A allele in gDNA of offspring was derived from either sow or boar. Subsequently, cDNA from six tissues including heart, kidney, liver, lung, muscle and spleen of offspring were sequenced to investigate whether or not allele-specific expression occurs. In all tested tissues of both offspring from two different combinations of boar and sow, allele-specific patterns were not shown and biallelic expressions with an approximate A:G ratio of 1:1 were detected (Figure 4).

### Comparative approach highlighted diversity and universality of CALCR and ASB4 imprinted cluster

**Figure 5:**
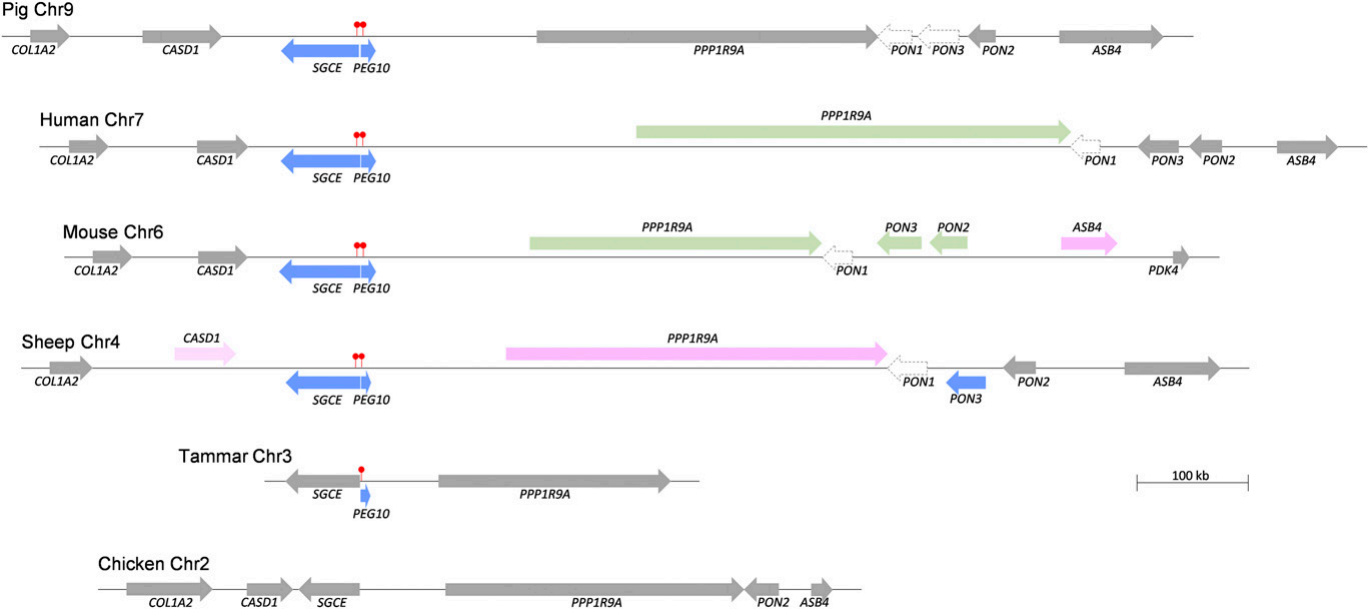
Simplified schematic representation of methylation and gene expression within the region spans between *COL1A2* and *ASB4* (or *PDK4*) genes in various species. *Filled red lollipops* indicate paternally hypermethylated DMRs. Paternal (*blue*), maternal (*pink*), weak maternal (*light pink*), biallelic (*gray*), tissue and/or stage-dependent maternal or biallelic (*green*) and low (*blank with dotted gray perimeter*) expressions are denoted. The direction of transcription is indicated with *arrows*. The genomic distance was scaled based on the NCBI gene database (http://www.ncbi.nlm.nih.gov/gene), except the tammar which was based on Suzuki *et al.*, 2007.

Imprinted expression of the genes located between *CALCR* and *ASB4* was compared between mice and humans (Table 2). In general, expression patterns in the porcine embryos within the syntenic region were closer to the expression in humans than that of mice, but in the pig the *PPP1R9A* gene showed biallelic expression in all tested tissues [embryonic and 2-day-old in the current study, and 2-month-old as reported in (Zhang *et al.* 2011)]. This biallelic expression of porcine *PPP1R9A* was different from the mouse and human which showed maternal expression in embryonic or fetal skeletal muscle tissues (Nakabayashi *et al.* 2004; Monk *et al.* 2008) and ovine fetuses that showed maternal expression in the kidney (Duan *et al.* 2018). Our polymorphism-based analysis revealed biallelic expression of *PPP1R9A* in the muscle and kidney in 2-day-old neonates (Figure 4). In addition, based on this study and literature, the imprinting pattern was illustrated by displaying differences and similarities among various species: selected mammals (pig, human, mouse, and sheep), the marsupial tammar wallaby with imprinting of *PEG10* but not *SGCE* (Suzuki *et al.* 2007), and the avian (chicken) deficient in imprinting (Frésard *et al.* 2014) (Figure 5).

**Table 2:** Biallelic and/or parental-origin-dependent expression of genes in the genomic region between *CALCR* and *ASB4* in mice and humans

	Mouse chr6A1			Human chr7q21.3		
*CALCR*	Bi: all embryonic tissues (E12.5 and 14.5) (except brain)	Sequencing	[1]	Bi: all fetal tissues (8-18 wk) (except brain)	Sequencing	[1]
	M: embryonic brain (E15.5), adult brain (Brain-specific)	RFLP	[2]	monoallelic: fetal brain	ns	[1]
*GNGT1*	Bi: embryonic tissues, placenta, yolk sac (E12.5 and 14.5)	ns	[1]	Bi: all fetal tissues (8-18 wk), placenta	Sequencing	[1]
*TFPI2*	Bi: embryonic tissues (E12.5 and 14.5)	Sequencing	[1]	absence of expression in fetus (8-18 wk)	RT-PCR	[1]
	M: placenta, yolk sac (E12.5 and 14.5)	Sequencing	[1]	M: placenta (polymorphic^†††^)	Sequencing	[1]
*GNG11*	Bi: embryonic tissues, placenta, yolk sac (E12.5 and 14.5)	ns	[1]	Bi: all fetal tissues (8-18 wk), placenta	Sequencing	[1]
*BET1*	Bi: embryonic tissues, placenta, yolk sac (E12.5 and 14.5)	ns	[1]	Bi: all fetal tissues (8-18 wk), placenta	Sequencing	[1]
*COL1A2*	Bi: embryo (all tested tissues, E15.5)	ns	[3]	Bi: all fetal tissues (8-18 wk), placenta	Sequencing	[1]
*CASD1*	Bi: embryo, placenta, yolk sac (E10 and 13)	RFLP	[4]	Bi: all fetal tissues (8-18 wk), placenta	Sequencing	[1]
	Bi: neonate (brain)	RFLP	[4]			
*SGCE*	P: embryo at E13	RFLP	[5]	P: patUPD7 cell line/ family	RT-PCR/ sequencing	[7]
	P: adult (brain, heart, kidney)	RFLP	[4]			
*PEG10*	P: embryo at E10	Sequencing	[4]	P: embryonic villi	RFLP	[8]
				P: patUPD7 cell line	RT-PCR	[7]
				P: fetus (8-18 wk), placenta	Sequencing	[1]
*PPP1R9A*	Bi: embryo (brain, liver, lung, eye) (E15.5) & neonate (brain)	Sequencing/ SNaPshot^†^	[6]	Bi: all fetal tissues (8-18 wk) except muscle	Sequencing	[1]
	M^p^: embryo (tongue), placenta, yolk sac & neonate (limb, tongue^††^)	Sequencing/ SNaPshot	[6]	M: fetal muscle (8-18 wk), 1^st^ trimester placenta	Sequencing	[1]
*PON1*	low expression in embryo, placenta, yolk sac	ns	[4]	undetectable (lymphoblasts)	ns	[9]
	Bi: Neonate (liver, lung)	RFLP	[4]			
*PON3*	M^p^: embryo (E10), placenta (E10) & yolk sac (E13)	RFLP	[4]	Bi: all fetal tissues (8-18 wk), placenta	Sequencing	[1]
	Bi: Neonate (liver, lung)	RFLP	[4]			
*PON2*	M^p^: placenta (E10) & yolk sac (E13)	RFLP	[4]	Bi: all fetal tissues (8-18 wk), placenta	Sequencing	[1]
	Bi: Neonate (all tested tissues)	RFLP	[4]			
*ASB4*	M: embryo at E15.5 (All tested tissues)	RFLP	[3]	Bi: fetus (8-18 wk) (liver, heart), placenta	Sequencing	[1]

*ns*: data not shown, *P*: paternal expression, *M*: maternal expression, *M^p^*: preferential maternal expression, *Monoallelic*: monoallelic expression (Parental origin was not determined due to absence of parental samples), *E*: embryonic day. ^†^Quantitative allele frequency measurement for SNPs. ^††^Limb & tongue represent skeletal muscle tissues, while placenta & yolk sac represent extra-embryonic tissues. ^†††^Some placenta showed maternal expression and others showed biallelic expression. [1] Monk *et al.* 2008; [2] Hoshiya *et al.* 2003; [3] Mizuno *et al.* 2002; [4] Ono *et al.* 2003; [5] Piras *et al.* 2000; [6] Nakabayashi *et al.* 2004; [7] Grabowski *et al.* 2003; [8] Ono *et al.* 2001; [9] Okita *et al.* 2003.

## Discussion

In this study, we report a comprehensive assessment of DNA methylation and allelic expression in the large chromosomal interval between *COL1A2* and *ASB4* in pigs. Our generation of parthenogenetic porcine embryos followed by strategies that combine whole-genome DNA methylation, transcriptome, and SNP-based allelic expression enabled the large-scale imprinting profiling on a genomic level. The methylome and transcriptome were constructed independently for three individuals in each control and parthenogenetic group to reduce genetic variability, and SNP analysis further evaluated allelic expression of the *PPP1R9A* gene in neonatal pigs. This experimental design was appropriate to detect, not only paternal expression which showed an all-or-none fashion (*i.e.*, *SGCE* and *PEG10* expressions in only control embryos having a paternal allele), but also allelic expression which was verified by the ensuing SNP analysis, followed by determining the boundary of imprinting cluster.

Genomic imprinting operates in mammals via several gene silencing mechanisms: *e.g.*, i) direct silencing of a promoters where the non-transcribed allele is heavily methylated, ii) inhibiting long-range communication between promoters and enhancers by binding of an insulator on the unmethylated allele and forming chromatin loops, iii) repressing a long noncoding RNA (ncRNA) transcript by methylation only on one of the parental alleles and silencing the other allele by the non-repressed long ncRNA. iv) isoform-dependent silencing originated from alternative promoter usage where different isoforms are silenced or active depending on the influence of DNA methylation (Barlow and Bartolomei 2014; Ahn *et al.* 2020). Regarding the third mechanism, unlike other known imprinted clusters, there was no evidence of a long ncRNA influenced by the porcine *SGCE*/*PEG10* DMR as reported in the mouse and human (Monk *et al.* 2008). Also, alternative promoter usage in the fourth mechanism was not likely to occur in this genomic interval because there was no evidence of expression of isoforms other than the displayed main transcripts according to our screening of transcriptome data using Integrative Genomics Viewer (IGV). Within the porcine *COL1A2-ASB4* region, maternal alleles of *SGCE*/*PEG10* locus appears inactive through direct silencing of promoters by maternal hypermethylation as described in the first mechanism, resulting in paternal expression. In addition, this maternal hypermethylation (and an insulator binding on unmethylated allele) could be involved in inhibition of long-range communication between a promoter and an enhancer as described in the second mechanism. However, genes other than *SGCE* and *PEG10* including *PPP1R9A* did not show an allele-specific expression pattern, but a biallelic expression, and therefore the insulator-mediated mechanism was not the mode of action in the *SGCE*/*PEG10* imprinting cluster in pigs. A previous study showed, using semiquantitative RT-PCR, expression of porcine *PPP1R9A* was greater in the parthenote than the control placental sample (a parthenote: control ratio of 1.7), suggesting maternal expression, but similar in the brain, fibroblast, and liver (Bischoff *et al.* 2009). Another study revealed that expression of *PPP1R9A* is biallelic in multiple tissues of 2-month-old piglets, except skeletal muscle, fat, and testis in which expression of *PPP1R9A* was not detectable (Zhang *et al.* 2011). To confirm whether or not allele-specific expression of *PPP1R9A* occurs in neonatal pigs, polymorphism-based assays were conducted for both parents and offspring and it revealed a biallelic expression of *PPP1R9A* which is different from its maternal expression in the mouse, human, and sheep (Ono *et al.* 2003; Nakabayashi *et al.* 2004; Duan *et al.* 2018).

The telomeric (distal) boundary of the large imprinted cluster organized around *Sgce*/*Peg10* locus in the mouse proximal region toward the centromere of chromosome 6 (6A1) reaches the *Asb4* gene, based on previous reports on maternally expression of *Ppp1r9a*, *Pon3*, *Pon2*, and *Asb4* in mice (Piras *et al.* 2000; Mizuno *et al.* 2002; Ono *et al.* 2003; Nakabayashi *et al.* 2004). However, the boundary spans between *CASD1* and *PON3* in sheep (Duan *et al.* 2018), while in the human it spans between *SGCE* and *PPP1R9A* (Ono *et al.* 2001; Grabowski *et al.* 2003; Okita *et al.* 2003; Monk *et al.* 2008). In addition, there are reports about monoallelic expression of mouse *Calcr* and *Tfpi2* and human *CALCR* (in brain) and *TFPI2* toward the centromeric (proximal) region, but the expression of those genes was restricted to brain and placenta, respectively (Hoshiya *et al.* 2003; Monk *et al.* 2008). Our porcine parthenogenetic embryos showed an imprinting boundary between *SGCE* and *PEG10* which is narrower than the human one, and expression of *CALCR* and *TFPI2* was not monoallelic in this study (Figure S1). As illustrated, genomic imprinting of *SGCE*/*PEG10* is conserved among mammals, but not in the marsupial and avian (Figure 5). The tammar wallaby showed a distinct imprinting pattern with a DMR located only at the promoter region of *PEG10*, but not within the promoter region of *SGCE* (Suzuki *et al.* 2007), while the chicken has been found to be deficient in genomic imprinting (Shin *et al.* 2010; Renfree *et al.* 2013; Frésard *et al.* 2014). Regarding the role of imprinting, both the murine *Sgce* and *Peg10* genes are expressed in the embryonic tissues and their paternal expression is essential for embryonic development, while maternal uniparental inheritance of the genomic region containing *Sgce* and *Peg10* is associated with embryonic lethality (Piras *et al.* 2000; Ono *et al.* 2006). As shown in *Peg10* knockout mice, deletion of *Peg10* gave rise to growth retardation and an early embryonic lethal phenotype (Ono *et al.* 2006). It has been reported that polymorphisms in *PEG10* genes are associated with carcass traits and fat deposition in pigs (Qiao *et al.* 2016), and *SGCE* and *PEG10* are candidate genes for piglet birth weight (Zhang *et al.* 2014). Taken together, genomic imprinting in the *SGCE-PEG10* cluster may play an important role in growth and development of pigs.

As shown in Table 2, parent-of-origin-dependent expression is often a developmental stage- and tissue-specific. In the mouse, embryonic days of analyzed embryos were between E10 and E15.5 which corresponds to Carnegie Stage (CS) 11-22, and the d21 porcine embryos used in this study were at CS15 (Hikspoors *et al.* 2016). Human fetal tissues collected in a previous study were from 8- to 18-week-old fetuses (which are in or after CS23) that were obtained at the termination of pregnancies (Monk *et al.* 2008). Therefore, the developmental stage of murine and porcine embryos was earlier than the human fetuses which requires careful attention. In addition, although our study did not include *in vitro* matured oocytes undergone *in vitro* fertilization (IVF), a previous study using these IVF oocytes showed that more exposure to *in vitro* culture condition might produce an adverse effect on dynamics of DNA methylation and increase DNA methylation during embryonic development (Deshmukh *et al.* 2011). This can suggest that there is a possibility of increased DNA methylation during *in vitro* handling even though our PA oocytes were transferred into the oviduct instead of culturing *in vitro* after activation. However, the degree of increase could be low, because in our previous reports almost complete absence of DNA methylation in PA was found in *Nesp* DMR which is paternally hypermethylated (Ahn *et al.* 2020). Additionally, there were some embryonic losses due to abnormal shaping but, among well-developed d21 PA embryos, we selected PA embryos that can be compared with control embryos based on intact morphology and sizes. Furthermore, the same chromosomal content was compared between control and PA embryos by genotyping the sex of control embryos and selecting female control embryos (*i.e.*, control embryos with 36,XX were compared with PA embryos with a double of 18,X).

In conclusion, porcine parthenogenetic embryos were used to explore a putative imprinted genomic interval, and this study revealed that the porcine *SGCE/PEG10* locus may not be clustered with a nearby gene locus. This cluster contains a single *cis*-acting DMR/ICR at the *SGCE*/*PEG10* locus, and direct silencing of promoters by hypermethylation is the mechanism underlying genomic imprinting of *SGCE/PEG10*. Our findings provide a comprehensive overview of one of the important imprinted clusters of pig chromosome 9 that may be implicated in the regulation of pig growth and development. Additionally, our dataset and forthcoming publication will greatly facilitate analyses on other imprinted loci in the pig genome.
